# Milestones in the systemic treatment of lung cancer

**DOI:** 10.1007/s12254-017-0313-9

**Published:** 2017-02-06

**Authors:** Robert Pirker

**Affiliations:** Department of Medicine I, Medical University of Vienna, Währinger Gürtel 18–20, 1090 Vienna, Austria

**Keywords:** Palliative chemotherapy, Adjuvant chemotherapy, Targeted therapy, Immune checkpoint inhibitors

## Abstract

Several milestones in the systemic treatment of lung cancer have been reached. Combination chemotherapy of small cell lung cancer was the first of these milestones. The establishment of palliative chemotherapy in patients with advanced non-small cell lung cancer and of adjuvant chemotherapy in patients with completely resected non-small cell lung cancer was another important milestone. Targeted therapies with angiogenesis inhibitors, EGFR inhibitors, and ALK inhibitors also advanced treatment. The clinical introduction of immune checkpoint inhibitors was the latest milestone.

## Introduction

Although a rare disease in 1900, lung cancer has become the most common cancer worldwide because of the smoking epidemic of the twentieth century. In 2012, 1.8 million patients were diagnosed with and 1.6 million patients died from this cancer [1]. While lung cancer has been classified as small cell lung cancer (SCLC) and non-small cell lung cancer (NSCLC) for many decades, the 2015 classification of lung cancer mandates further subclassification by means of immunohistochemical and molecular analyses in routine clinical practice [2]. Patients with advanced NSCLC, particularly those with adenocarcinomas, are currently tested for the presence of epidermal growth factor receptor (EGFR) mutations, ALK aberrations, and ROS1 aberrations in their tumors because these molecular alterations have therapeutic consequences. Additional molecular analyses are expected to enter routine clinical practice in the near future.

Several therapeutic advances have occurred over the years. Major milestones in the treatment of lung cancer were combination chemotherapy for SCLC, palliative chemotherapy for advanced NSCLC, adjuvant chemotherapy in patients with completely resected NSCLC, targeted therapies, and, most recently, treatment with immune checkpoint inhibitors. The present manuscript summarizes these therapeutic milestones.

## Combination chemotherapy for SCLC

SCLC is often widespread with clinically detectable metastases in about two thirds of the patients at the time of diagnosis. SCLC has been recognized as a chemosensitive disease and the introduction of combination chemotherapy in the second half of the last century was unequivocally a milestone in the treatment of lung cancer (see [3] for review). Palliative combination chemotherapy increases median survival times four- to five-fold compared with best supportive care alone and relieves cancer-related symptoms in the majority of symptomatic patients. Combination chemotherapy was shown to be superior to single agents in terms of survival and quality of life, thereby stressing the fact that tumor control by chemotherapy clearly outweighs the toxicities associated with combination chemotherapy [3]. Further therapeutic advances were the introduction of thoracic radiotherapy in patients with limited disease, prophylactic brain irradiation in patients who have responded to initial therapy, and topotecan as second-line chemotherapy.

## Palliative chemotherapy of advanced NSCLC

The establishment of palliative chemotherapy in patients with advanced NSCLC, particularly in the first-line setting, is considered as a therapeutic milestone. A meta-analysis of randomized trials was published in 1994 and demonstrated a survival benefit from first-line chemotherapy compared with best supportive care alone [4]. This led to the widespread implementation of first-line chemotherapy as clinical standard for patients with advanced NSCLC.

Further improvements were achieved by the third-generation cytotoxic drugs, which were shown to be superior and/or better tolerated than older drugs. Currently, patients receive first-line chemotherapy with up to six cycles of a platinum-based doublet containing a third-generation cytotoxic drug [5, 6]. Compared with best supportive care alone, first-line chemotherapy increases median survival by 1.5 months and the 1‑year survival rate by 9% and also relieves cancer-related symptoms in about one half of the symptomatic patients [5].

Cisplatin-based chemotherapy is slightly superior to carboplatin-based chemotherapy [7]. Cisplatin-based chemotherapy resulted in a higher response rate compared with carboplatin-based chemotherapy (30% vs. 24%) and carboplatin-based chemotherapy was associated with an increase in mortality in patients treated with third-generation anticancer drugs (HR = 1.11; 95% confidence interval = 1.01–1.21) and also in patients with non-squamous NSCLC (HR = 1.12; 95% confidence interval = 1.01–1.23) [7]. In clinical practice, cisplatin-based chemotherapy is preferred in patients with good performance status in the absence of clinically relevant comorbidities, whereas carboplatin-based chemotherapy is used in patients with reduced organ functions (kidney, heart) and when ease of administration is of particular importance. Elderly patients and patients with reduced performance status also benefit from well-tolerated chemotherapy protocols such as single agents or carboplatin-based doublets.

First-line chemotherapy is currently combined with bevacizumab in selected patients with non-squamous cell NSCLC or with necitumumab in patients with EGFR-positive squamous cell NSCLC (see next section). After the establishment of first-line chemotherapy, maintenance therapy and second-line therapy became standard treatment for selected patients with advanced NSCLC [6].

## Adjuvant chemotherapy in patients with resected NSCLC

Approximately 25–30% of patients with NSCLC are diagnosed with localized disease and undergo surgery with curative extent. Up to 70% of these patients, however, will relapse systemically because of the presence of micro-metastases at the time of surgery. Therefore, adjuvant chemotherapy was studied in order to improve the outcome of patients with completely resected NSCLC. The meta-analysis of early randomized trials published in 1994 suggested a trend toward improved survival for adjuvant chemotherapy [4]. This potential survival benefit together with the availability of better anticancer drugs and anti-emetics led to the re-evaluation of adjuvant chemotherapy in randomized trials on large patient populations.

Three of these randomized trials demonstrated a survival benefit for adjuvant cisplatin-based chemotherapy in patients with completely resected NSCLC [8–11]. The increase in the 5‑year survival rates ranged between 4 and 15% in these trials. The Lung Adjuvant Cisplatin Evaluation (LACE) meta-analysis, which was based on 4,584 patients from five cisplatin-based chemotherapy trials (ALPI-EORTC, IALT, JBR10, ANITA, Big Lung Trial), confirmed the survival benefit with a hazard ratio of 0.89 (95% CI = 0.82–0.96; *p* = 0.004) for adjuvant chemotherapy which translates into a survival benefit of 5.3% ± 1.6% at 5 years [12]. Disease-free survival was also prolonged by adjuvant chemotherapy and the hazard ratio was 0.8 (95% CI = 0.78–0.9; *p* < 0.001). These findings led to the establishment of adjuvant cisplatin-based chemotherapy as a standard for patients with completely resected NSCLC stages II and III and for selected patients with stage IB. Adjuvant chemotherapy consists of four cycles of a cisplatin-based doublet. Based on the evidence from clinical trials, cisplatin plus vinorelbine can be considered as the preferred chemotherapy protocol.

Strategies that have been studied to improve the outcome of adjuvant chemotherapy include the characterization of predictive biomarkers for patient selection, customized chemotherapy based on biomarkers, targeted therapies, and immunotherapies. To date, however, none of these strategies has been successful.

## Targeted therapies

The establishment of targeted therapies in patients with advanced NSCLC is another therapeutic milestone. Targeted therapies have focused on inhibition of angiogenesis, EGFR, or ALK.

While many angiogenesis inhibitors have been studied, only few have entered clinical practice. Bevacizumab was the first angiogenesis inhibitor to be approved in combination with platinum-based chemotherapy in patients with advanced non-squamous NSCLC. This approval was based on the results of two phase 3 trials that demonstrated superior outcome for chemotherapy plus bevacizumab compared with chemotherapy alone in patients with advanced non-squamous cell NSCLC [13, 14].

Nintedanib, a triple kinase inhibitor, and ramucirumab, a monoclonal antibody against vascular EGFR, have also been approved in the combination with second-line chemotherapy with docetaxel. Compared with docetaxel alone, nintedanib added to docetaxel improved progression-free survival and, in patients with adenocarcinomas, also overall survival [15]. This led to the approval of nintedanib in combination with docetaxel for patients with advanced adenocarcinomas who have been pretreated with chemotherapy. Ramucirumab increased survival when added to docetaxel in patients with advanced NSCLC and was also approved [16].

Blockade of the EGFR by monoclonal antibodies, tyrosine kinase inhibitors, and other approaches has also been studied to improve outcome in patients with advanced NCSLC (for review, see [17]). Several anti-EGFR monoclonal antibodies have been evaluated in clinical trials (for review, see [18]). First-line chemotherapy combined with cetuximab improved survival in patients with advanced NSCLC, particularly in those with high EGFR expression in their tumors [19, 20]. This benefit was confirmed in a meta-analysis based on four randomized trials [21]. Necitumumab added to first-line chemotherapy with cisplatin plus gemcitabine increased survival of patients with advanced squamous cell NSCLC [22]. Although the benefit with cetuximab was similar to the one of necitumumab, only necitumumab has been approved by the European Medicines Agency.

Tyrosine kinase inhibitors of the EGFR have initially been studied in unselected patients with advanced NSCLC. Excellent activity of these drugs was seen in never-smokers, patients of South-East Asian ethnicity, and patients with adenocarcinomas. In 2004, these patients were found to harbor EGFR mutations in their tumors [23–25]. Several studies then demonstrated the superiority of tyrosine kinase inhibitors over first-line chemotherapy in terms of progression-free survival and quality of life in patients with advanced EGFR mutation-positive NSCLC (for review, see [17]). A survival benefit has been demonstrated for afatinib [26]. Thus patients with advanced EGFR mutation-positive NSCLC receive EGFR tyrosine kinase inhibitors as first-line therapy. At the time of disease progression, patients are switched to chemotherapy or, in the presence of the T790 M mutation, to osimertinib [27, 28]. Patients with advanced ALK-positive NSCLC are treated with crizotinib or other ALK inhibitors [29–31].

## Immune checkpoint inhibitors

The most recent milestone in the treatment of lung cancer was the introduction of immune checkpoint inhibitors into clinical practice in patients with advanced NSCLC. These drugs are directed against cytotoxic T lymphocyte-associated antigen 4 (CTLC4), programmed death-1 (PD-1), and PD-1 ligands PD-L1 and PD-L2. Drugs that have been studied in lung cancer are ipilimumab, nivolumab, pembrolizumab, atezolizumab, durvalumab, and avelumab.

Nivolumab and pembrolizumab have shown efficacy in phase 3 trials in patients with advanced NSCLC who had been pretreated with chemotherapy [32–34]. Nivolumab has shown a survival benefit compared with docetaxel in advanced squamous NSCLC and in adenocarcinomas [32, 33]. Pembrolizumab showed superiority over docetaxel in patients with PD-L1 expression on at least 1% of tumor cells [34]. The survival benefit was seen with both doses of pembrolizumab (hazard ratios of 0.71 and 0.61 for 2 mg/kg and 10 mg/kg, respectively) and was more pronounced in patients with PD-L1expression in 50% or more of tumor cells. The anti-PD-L1 antibody atezolizumab improved overall survival compared with docetaxel in a randomized phase 2 trial, particularly in patients with enriched PD-L1 expression on tumor cells and tumor-infiltrating immune cells [35]. Side effects of immune checkpoint inhibitors are immune-related pneumonitis, colitis, hepatitis, nephritis, endocrinopathies, infusion-related events, and others [31–35].

Immune checkpoint inhibitors have also been studied in the first-line setting. Pembrolizumab has demonstrated superior progression-free survival and overall survival compared with platinum-based chemotherapy in patients with PD-L1 expression in at least 50% of the tumor cells [36]. By contrast, nivolumab failed to improve outcome compared with chemotherapy in patients with PD-L1 expression in at least 5% of tumor cells [37].
